# Editorial: Fetal-maternal monitoring in the age of artificial intelligence and computer-aided decision support: A multidisciplinary perspective

**DOI:** 10.3389/fped.2022.1007799

**Published:** 2022-09-05

**Authors:** Antoniya Georgieva, Patrice Abry, Ines Nunes, Martin G. Frasch

**Affiliations:** ^1^Nuffield Department of Women's and Reproductive Health, University of Oxford, Oxford, United Kingdom; ^2^CNRS, École Normale Supérieure de Lyon, Laboratoire de Physique, Lyon, France; ^3^Centro Materno Infantil Do Norte–Centro Hospitalar Universitário Do Porto, Porto, Portugal; ^4^Centro Académico Clínico, Instituto de Ciências Biomédicas Abel Salazar, Centre for Health Technology and Services Research, Faculty of Medicine, University of Porto, Porto, Portugal; ^5^Department of Obstetrics and Gynecology and Center on Human Development and Disability, University of Washington, Seattle, WA, United States

**Keywords:** electronic fetal monitoring, cardiotocography, electrocardiography, electroencephalography, pregnancy, machine learning, decision support, big data and analytics

## Introduction

Across the globe, each day, we continue to have term babies arrive at delivery wards in good condition *in utero*, only to be born hours later with neurological injuries (1). The consequences are profound and life-long for the babies, parents, siblings, and their wider family (2). Clinical staff involved in the obstetric management are severely impacted in multiple ways. On the other hand, Cesarean section to avoid oxygen deprivation during labor carries multiple risks for mother, fetus, future pregnancies; as well as costs. But achieving safe spontaneous delivery is sometimes challenging due to poorly understood and complex fetal physiology, and often, conflicting healthcare needs for mother and baby.

In developed countries, the standard of care for pregnancies deemed at risk is continuous electronic fetal monitoring with cardiotocography (CTG) during labor—displaying fetal heart rate (FHR) along uterine contractions on a long paper strip, typically assessed by eye. CTG interpretation continues to be a massive challenge with high false positive rate and poor sensitivity (3). The evidence how to interpret CTG traces is relatively limited, derived by subjective clinical experience and by animal studies, which have own limitations, but provide invaluable core evidence (4–7). For example, there has been an ongoing concern about the persistent use of “increased” or “decreased” heart rate variability (HRV) which goes against the evidence of what variability represents—a complex dynamic multi-dimensional pattern, such that a single-order description of this pattern in terms of “ups” and “downs” discards most of its predictive information (8). Furthermore, the CTG technology itself is imperfect with frequent signal loss, noise, and confusion between maternal and fetal heart rates (3).

So, the main challenge remains lack of technology to monitor reliably the fetus *in utero*, at a time when labor brings unprecedented challenges to fetal oxygen supply and forces the fetus to rely on compensatory mechanisms and reserves. Consequently, the adverse outcomes we wish to prevent are both heterogeneous and rare. This means that, to develop new detection/prediction methods or algorithms based on the CTG and utilize our modern computing and data science capabilities, we need to obtain CTG and maternity data at scale (9). And there is a need of “feature engineering”, i.e., bespoke signal processing methods to account for the noisy, low sampling rate nature of the signal (10). Despite these known shortcomings and promising opportunities to make considerable impact on human health, improving fetal monitoring through novel technologies continues to be a niche field, especially for large scale clinical use. It remains unclear how to best apply computers and large datasets for clinical benefit, hand-in-hand with novel engineering solutions.

Therefore, we assembled this first of its kind Frontiers Research Topic, focused on multidisciplinary intrapartum risk assessment through technology and clinical insights. It builds on our experience and existing collaboration in organizing the bi-annual international workshop—Signal Processing and Monitoring in Labor—providing multidisciplinary forum for the clinical and engineering challenges of fetal monitoring during labor. These workshops have included experts from academia and industry representing multidisciplinary domains of clinical medicine (obstetrics, neonatology), physiology, physics, epidemiology, data sciences, statistical signal processing, artificial intelligence (AI), and signal feature and software engineering. We hope that the multi- and transdisciplinary character of these workshops can serve as a template for the framework in which solutions to the problem of fetal monitoring intrapartum can be found. In the following, we synthesize the core insights provided by the 15 contributions (four systematic reviews, one opinion and ten original research articles) of this Research Topic. We then discuss the future directions for this field.

## Systematic reviews

O'Sullivan et al. review the decision support systems used in three RCTs for intrapartum CTG, summarizing the algorithms, the outcomes of the trials and the limitations (O'Sullivan et al.). Preliminary work suggests that the inclusion of clinical data can improve the performance of AI-assisted CTG. Combined with newer approaches to the classification of CTG traces, this offers promise for rewarding future development.

Castel et al. screened 256 studies in four languages and arrived at 40 studies in the qualitative and quantitative analysis of the intrapartum fetal electroencephalogram (fEEG). The authors show its potential to act as a direct biomarker of fetal brain health during delivery, ancillary to FHR monitoring and readily feasible using the presently used fetal scalp electrode. Real world evidence of fetal EEG acquired from a regular fetal scalp electrode is also presented. Highlighted is the need for clinical prospective studies to further establish the utility of intrapartum fEEG monitoring intrapartum, suggesting suitable clinical study designs.

Ribeiro et al. focused on non-linear analysis of FHR based on concepts of chaos, fractality, and complexity: entropies, compression, fractal analysis, and wavelets. The authors aim to increase our knowledge about cardiovascular dynamics in healthy and pathological fetuses. Two hundred and seventy articles are included in the review. The top five primary research objectives covered by the selected papers are detection of hypoxia, maturation or gestational age, intrauterine growth restriction, and fetal distress.

This review shows that non-linear indices can be used but are not yet applied in clinical practice. Some studies show that the combination of several linear and non-linear indices would be ideal for improving the analysis of the fetal wellbeing. Future studies should narrow the research question so a meta-analysis could be performed, probing the indices' performance.

Castro et al. review the spectral bands reported in intrapartum FHR studies and evaluate their performance in detecting fetal acidemia. Twenty-five (out of 176) studies are included. An open-access FHR database is used, with recordings of the last half an hour of labor of 246 fetuses. Four different umbilical artery pH cut-offs are considered for fetuses' classification into acidemic or non-acidemic: 7.05, 7.10, 7.15, and 7.20. The area under the receiver operating characteristic curve (AUROC) is used to quantify the frequency bands' ability to distinguish acidemic fetuses.

Bands referring to low frequencies, mainly thought to be associated with neural sympathetic activity, are found to be the best at detecting acidemic fetuses, with the more severe definition (pH ≤ 7.05) attaining the highest values for the area under the ROC–AUROC [0.770 (0.608–0.932)].

This study shows that the power spectrum analysis of the FHR is a simple and powerful tool that has potential for CTG evaluation and helping healthcare professionals to accurately identify fetuses at risk of acidemia. Naturally, prospective clinical evaluations are needed. Despite the clear potential of many HRV metrics to predict fetal acidemia, the proxy of fetal acidemia itself has been proven to not be appropriate for predicting clinical outcome (11, 12). As such, while to date, most studies in the field have sought to predict pH at birth, some studies in this Research Topic and elsewhere have sought to predict physiologically more direct outcomes related to fetal compromise, either using FHR data as time series or as scanned CTG tracings (Gold et al.; Roux et al.).

In this context, the opinion paper by Schifrin provides insights into the complexities of CTG interpretation that transcend the conventional scientific reasoning applied in this field and extend to historical and medicolegal confines as well as epistemological biases. The author emphasizes the problem of the poor definition of outcome, centered on pH prediction and avoidance of low pH while providing little guidance to intrapartum management. He discusses opportunities for future research which, in part, are tackled in the above-mentioned reviews, as well as in the research articles of this topic we discuss in the following paragraphs.

## Original contributions

It has been striking to observe that all studies in the present Research Topic relied on retrospective data analysis. It highlights the major issue in our field which impedes rapid innovation: the data required to test models predicting adverse outcome with clinically actionable performance must be large (millions of CTGs) and no single institution, company or research team have access to such data at this time.

Several studies deal with the signal quality issues which also hamper the progress in the field. While some approaches seek to improve the ultrasound-based CTG signal and its derived HRV estimates by thoughtfully engineering signal processing and machine learning techniques (Roux et al.; Vargas-Calixto et al.), others focus on the emerging technologies such as transabdominal ECG (Fotiadou and Vullings; Vullings and van Laar). It is worth noting that both EEG, reviewed by Castel et al., and transabdominal ECG technologies have been studied for at least 80 and 40 years, respectively. However, these technologies have not yet found a broad clinical adoption.

Pursuing further the issue of FHR signal quality in conventional CTG, Vullings and van Laar conduct a quantitative comparison of FHR derived from two commercial intrapartum ECG-based fetal monitors, one using fetal scalp electrode and another using transabdominal ECG sensors. The authors compare the FHR detection rates to those from a conventional ultrasound-based CTG. They report a reliable FHR in >95% of time intrapartum which represents a substantial improvement over Doppler ultrasound. During second stage of labor, given stronger contraction and FHR drops to below 100 bpm, the signal processing challenges remain considerable and the performance of the method decreases. However, with a reliability higher than 80%, the proposed method still outperforms Doppler ultrasound and other reference methods by a significant amount.

Vargas-Calixto et al. deploy a signal processing approach to identify robustness of select HRV estimates to noise contained in conventional ultrasound-based FHR. Such insights hold promise of improved fidelity of HRV estimation given the constraints of CTG signal in terms of quality of beat-to-beat estimation and noisiness. Their work highlights the importance of considering the nature of the underlying FHR signal when selecting and trusting the HRV metrics derived from the signal.

Fotiadou and Vullings turn their attention to the alternative approach of FHR derivation promising superior signal quality and patient experience compared to conventional ultrasound methods, the transabdominal ECG recorded antepartum and intrapartum. The authors present a method to extract the entire multi-channel fetal ECG waveform using deep convolutional neural networks (CNN), a broadly used deep learning technique for tasks such as image analysis and here showing promise for maternal-fetal ECG deconvolution. Meanwhile, in Frasch et al., CNN was also used to detect pathognomonic CTG patterns directly from images. This highlights the broad potential of deep learning techniques in various settings of fetal monitoring.

Costa et al. report the performance of their intrapartum CTG system SisPorto, focusing on prediction of pathological FHR patterns from hitherto underutilized HRV fragmentation metrics using the open-source Brno/Prague CTG database. In another retrospective study, Lovers et al. used computerized methods to analyze CTGs from ~28,000 births and identify presence of abnormalities in the first hour CTG as well as associated clinical risk factors. This highlights the importance of admission CTG analysis for labor management/triage. Another facet of this study is the indication of the importance of antepartum fetal health that likely precipitates the abnormalities seen in the admission CTG. The creation of maternal-fetal monitoring technologies that track health of mother-fetus dyad antepartum is much needed and subject of ongoing research and development.

In addition, Pini et al. contribute to the detection of late intrauterine growth restriction (IUGR) in a retrospective case-control design at 38 weeks of gestation, on a publicly available dataset of CTGs (Pini et al.). The HRV feature engineering the authors present for their machine learning model accounts for the CTG's properties such as signal duration and signal quality. Future studies should attempt to validate these findings in larger datasets and in admission CTGs to mimic the clinical scenario of intrapartum triaging at admission.

In their complementary work, Gold et al. and Roux et al. study a fetal sheep model-based FHR dataset of umbilical cord occlusions (UCO) of increasing severity mimicking the uterine contractions as they may occur during first and second stage of human labor. Both teams engineer novel machine learning techniques for prediction of a physiologically and clinically meaningful outcome of fetal cardiovascular decompensation, rather than acidemia, and to address the complex nature of fetal HRV signal accounting for the constraints on signal quality in terms of sampling rate and noise. Both studies present novel individualized approach to machine learning of FHR data. This is a promising avenue to exploit in future studies, even when the data size is relatively small, as it leverages individual variability at different time scales, to identify departure from the “normal” phenotype. And, using the same experimentally derived dataset, Rivolta et al. present novel HRV features of deceleration reserve as a distinguishing property of chronically hypoxic fetuses.

Finally, in their study on scanned CTG recordings, Frasch et al., for the first time, deploy computer vision techniques of deep learning, a form of AI, to identify important patterns of CTG on images, rather than the raw data. Importantly, the authors open-sourced their algorithm including the approach to annotate online the CTG data. This could potentially lead to better collaboration, for example crowdsourced CTG annotation.

## Conclusions and outlook for the future

Intrapartum fetal monitoring is, hopefully, on the verge of technological disruption thanks to the recent advances in and convergence of computing resources on edge and in the cloud, AI and the resulting emergence of digital health as a field. The clinical need remains unmet and, more than ever the chasm, between technological advances and possibilities and the reality of fetal monitoring around the world is wide and asking for closure. Thankfully, the technological disruption through innovative devices and algorithms is being pursued in Europe and USA as we have seen from the academic and industry partners in this Research Topic, as well as in the relevant Signal Processing and Monitoring Workshops in 2019 (Porto, Portugal) and the most recent 2022 workshop in Munich (Germany). There is a strong consensus across the board that the challenge of intrapartum fetal monitoring can only be solved through collective, multinational effort. The big data (millions of CTGs) required is impossible to collect by any single stakeholder. In parallel, physiological research needs to continue to address the fundamental questions raised in this Research Topic and elsewhere about the mechanisms of injury. Also required is the push for innovative technologies that can acquire other important signals for the fetus, i.e., ECG, EEG, and beyond.

**Figure 1:**
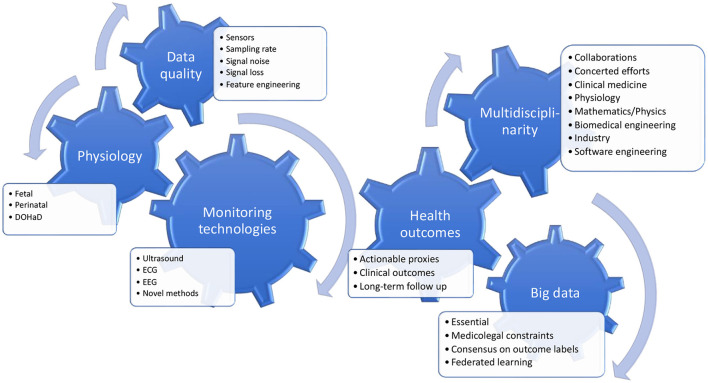
Key insights from the Research Topic and future directions. There is a growing awareness of the antecedents of intrapartum fetal reserve for the trial of labor which require an integration of the physiology of whole pregnancy and the well-known relationships between intrauterine adversity on one hand, and the perinatal and postnatal developmental trajectories on the other hand (Developmental Origins of Health and Disease, the DOHaD concept). Another key insight is the requirement for clinically actionable outcome labels in the prediction models that are being developed and the recognition of the fundamental constraints on the data sizes of the individually accessible cohorts. Therefore, there is a clear need for multinational and multidisciplinary work to address the different challenges and research questions, which are all integral to successfully improving the technologies for intrapartum fetal monitoring.

As the challenge is enormous, for a proposed shared partnership effort to succeed, we need to form a cohesive view of the shared direction. In Figure 1, we present such a cohesive view of the shared and interacting priorities, representing also our main take-home message from this Research Topic.

## Author contributions

AG and MF prepared the first draft of the manuscript. All authors edited and approved the final version.

## Funding

Author AG was funded by the UK National Institute of Health and Care Research (CDF-2016-09-004 and NIHR202117).

## Conflict of interest

Author MF holds patents on EEG and ECG processing, the founder of and consults for Digital Health companies commercializing the predictive potential of physiological time series for human health. The remaining authors declare that the research was conducted in the absence of any commercial or financial relationships that could be construed as a potential conflict of interest.

## Publisher's note

All claims expressed in this article are solely those of the authors and do not necessarily represent those of their affiliated organizations, or those of the publisher, the editors and the reviewers. Any product that may be evaluated in this article, or claim that may be made by its manufacturer, is not guaranteed or endorsed by the publisher.

## Author disclaimer

The views expressed are those of the author(s) and not necessarily those of the NIHR or the Department of Health and Social Care.
